# Long-Term Effect on Bioactive Components and Antioxidant Activity of Thermal and High-Pressure Pasteurization of Orange Juice

**DOI:** 10.3390/molecules23102706

**Published:** 2018-10-20

**Authors:** Fabiana N. Vieira, Sónia Lourenço, Liliana G. Fidalgo, Sónia A. O. Santos, Armando J. D. Silvestre, Eliana Jerónimo, Jorge A. Saraiva

**Affiliations:** 1 QOPNA, Department of Chemistry, University of Aveiro, 3810-193 Aveiro, Portugal; fabiananv@gmail.com (F.N.V.); sonia.lourenco.11.27@gmail.com (S.L.); jorgesaraiva@ua.pt (J.A.S.); 2 CICECO-Aveiro Institute of Materials, Department of Chemistry, University of Aveiro, 3810-193 Aveiro, Portugal; armsil@ua.pt; 3 Centro de Biotecnologia Agrícola e Agro-Alimentar do Alentejo/ Instituto Politécnico de Beja (IPBeja), 7801-908 Beja, Portugal; eliana.jeronimo@cebal.pt

**Keywords:** antioxidant activity, bioactive compounds, orange juice, high pressure, refrigerated storage, thermal processing, phenolic compounds

## Abstract

The long-term effect of thermal pasteurization (TP) and high-pressure processing (HPP) of orange juices stored under refrigeration, on the bioactive components and antioxidant activity, was compared. Total phenolic content (TPC), flavonoid, anthocyanin, and carotenoid contents, the individual content of major phenolic components, and the antioxidant activity, were evaluated in TP- and HPP-treated juices over a 36-day period. At day 0, no significant differences in TPC, and a decrease in carotenoid content after both treatments, were observed. TP caused a decrease of flavonoid and anthocyanin contents, while HPP increased flavonoid content. Three major phenolic components were identified: apigenin-6,8-di-*C*-glucoside, naringenin-7-*O*-rutinoside, and hesperetin-7-*O*-rutinoside, the latter increasing *ca.* 45% immediately after HPP. During storage, a decrease in TPC, and in the anthocyanin and carotenoid contents of both treated juices was observed, with higher anthocyanin and phenolic contents in HPP juices. A significant increase of hesperetin-7-*O*-rutinoside content was observed in HPP juice. Both treatments caused a decrease (26% and 13%, respectively) of antioxidant activity. Most of the kinetic profiles followed zero-order patterns, with HPP juices showing a considerably higher half-life than TP ones. These results clearly demonstrate the advantages of HPP for orange juice preservation allowing, also, their nutritional benefits to be enhanced by increasing the content of some bioactive components.

## 1. Introduction

In recent decades, the impact of human diet and the composition of food ingredients on human health has been the focus of increasing attention [1,2]. It is known that food nutritional value is highly dependent on its processing and, therefore, the demand for processed foods where health-promoting components are preserved, or containing added ingredients with a specific body function, named functional foods [3], has led to the development of new minimal-processing technologies that allow a better preservation of these components [3,4,5]. 

Heat treatments, such as pasteurization and sterilization, are the most used methods to process and preserve food, mainly due to their ability to inactivate a wide range of microorganisms and spoilage enzymes [6]. However, heat processing may induce several chemical and physical changes, reducing the content and, also, the bioavailability of some bioactive compounds [7,8]. In the search for new processing methods, a few non-thermal techniques have emerged. Pulsed electric fields (PEF), high-intensity light pulses, magnetic fields, and high-pressure processing (HPP) are currently among the most studied [9,10,11]. HPP has shown to be effective reducing microbial populations and preserving sensory qualities, without affecting the structure of small molecules, such as some bioactive compounds [9,12]. This technique uses pressure, up to 600 MPa, to inactivate some harmful and pathogenic microorganisms and enzymes responsible for the quality loss of food products, including several fruit juices [13]. 

Beyond vitamin C, orange juice is also one of the main sources of carotenoids (xanthophylls, cryptoxanthins, and carotenes), antioxidant flavanones (hesperetin and naringenin) and their glycosides, and other beneficial phytochemical compounds, such as folate [14,15,16]. Being one of the most consumed juices worldwide, it is well known that its organoleptic properties and nutritional value are deeply affected by conventional processing techniques [17]. Therefore, it is essential to have a deep understanding of which compounds are affected, and through which mechanisms, both during processing and long-term storage. In fact, the considerable decrease of phenolic compounds and vitamin C of orange juice during storage, leading also to a decrease in the juice antioxidant capacity, is well known [18,19]. Different studies have already evaluated the effect of HPP or thermal pasteurization (TP) in the composition and antioxidant activity of orange juices [18,20,21]. However, only a single study compared the long-term effect of the use of both techniques in the processing of orange juice [22], and only the effects on carotenoid and flavonoid contents were evaluated. In fact, the degradation kinetics of phenolic compounds of orange juice, during storage, is shown to be highly affected by the processing method used [23]. 

In this vein, the present study aimed to evaluate and compare the long-term effect of HPP and TP orange juices stored under refrigeration on the bioactive components content and antioxidant activity. Total phenolics, anthocyanins, flavonoids, and carotenoids were analyzed, in both HPP and TP orange juice, during 36 days of storage. The major component contents were also evaluated by high-performance liquid chromatography, equipped with a diode array detector and coupled to ion trap mass spectrometry (HPLC-DAD-MS^n^). In addition, the antioxidant activity was analyzed to understand the shelf-life effect on the quality of orange juice. The kinetic analysis of the degradation of phenolic compounds, flavonoids, anthocyanins, carotenoids, and antioxidant activity of TP and HPP orange juice was performed, allowing the comparison of the shelf-life of TP and HPP juices. Therefore, this study will be of particular relevance for the selection of emerging technologies, such as HPP, in the processing of orange juice, maximizing their nutritional and sensory quality.

## 2. Results

### 2.1. Effect of Storage of TP and HPP Orange Juice on Total Phenolic Content

**Figure 1:**
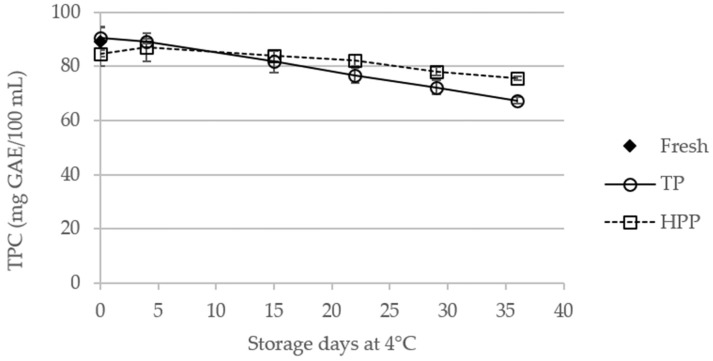
Total phenolic content (TPC) of fresh and thermal pasteurized (TP) and high-pressure processed (HPP) orange juices during storage at 4 °C.

The effect of TP and HPP on the TPC of stored orange juices was studied over 36 days, as shown in Figure 1. No significant changes were observed between the TPC of fresh and processed orange juices on the day of treatment (day 0). The statistical data are available in Supplementary Material (Table S1). The obtained TPC values are in the same range of those reported in the literature for orange juice [21,24]. During storage, the TPC of both TP and HPP orange juices decreased, this behavior being more significant in TP juices, for which a decrease of about 25% (*p* < 0.05) was observed after 36 days. In fact, a slight (but significant) difference was observed between the TPC of fresh juice (89.0 ± 5.4 mg gallic acid equivalents (GAE)/100 mL) and the TPC of HPP juice after 36 days of storage (75.7 ± 0.7 mg GAE/100 mL). Comparing the TPC of TP and HPP juices, a significant difference between them is only observed after 36 days of storage.

### 2.2. Effect of Storage of TP and HPP Orange Juice on Total Flavonoid Content

**Figure 2:**
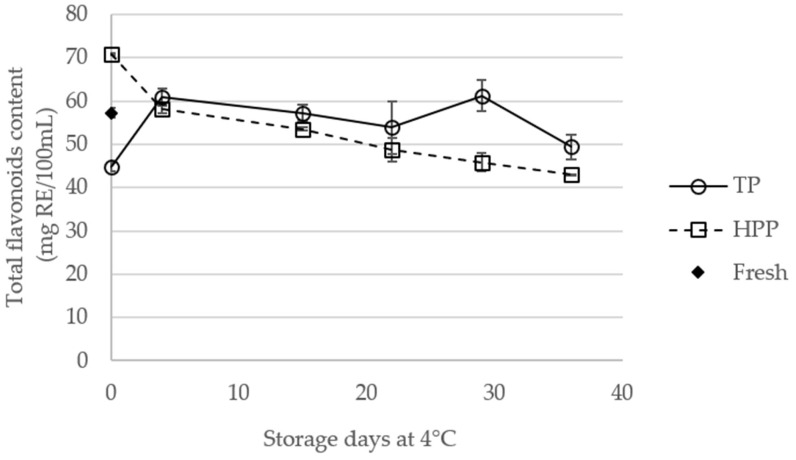
Total flavonoid content (expressed as rutin equivalents (RE)) of fresh and thermal pasteurized (TP) and high-pressure processed (HPP) orange juices during storage at 4 °C.

The total flavonoid content of fresh juice and processed orange juices during storage is shown in Figure 2. The flavonoid content of fresh and treated juices, at day 0, are considerably higher than those described in the literature for fresh or processed orange juices [16,21]. After treatment (day 0), HPP orange juices showed significantly (*p* < 0.05) higher flavonoid content compared to fresh and TP juices (the statistical data is showed in Supplementary Materials (Table S2)). Compared to fresh juice, HPP led to an increase of flavonoid content from 57.3 to 70.8 mg of rutin equivalents (RE)/100 mL (~24%). In fact, it has been suggested that HPP induces the decrease of cloud fraction particles, promoting the release of phenolic compounds, including flavonoids [21]. On the other hand, the TP juice presented the lowest flavonoid content at day 0, with a decrease of about 22% compared to fresh juice. Actually, the low stability of flavonoids on orange juices, submitted to thermal pasteurization (TP) processes, has been already reported [20,21].

The different evolution of flavonoid content in TP and HPP juices, during storage, showed that these treatments have distinct effects during long-term storage. A significant increase (*p* < 0.05), from 44.8 ± 1.0 to 60.8 ± 2.0 mg RE/100 mL, was observed for TP juices in the first 4 days of storage. It is possible that during storage some compounds are formed that will react with the aluminum chloride, increasing the total flavonoid content. From the 4th to 22nd day, the total flavonoid content in TP orange juice was not significantly affected. From the 22th to 29th day of storage, an increase (*p* < 0.05) was observed, after which the flavonoid content decreased (about 19%). Concerning HPP juices, a significant decrease of the total flavonoid content was observed during storage. This is in agreement with that reported in the literature for flavanones, and may be related with the residual activity of polyphenol oxidase (PPO) and peroxidase (POD) [22]. In addition, TP juice showed higher flavonoid content than HPP orange juices after 36 days of storage, indicating that HPP could compromise the flavonoid content in a long-term context.

### 2.3. Effect of Storage of TP and HPP Orange Juice on Total Anthocyanin Content

**Figure 3:**
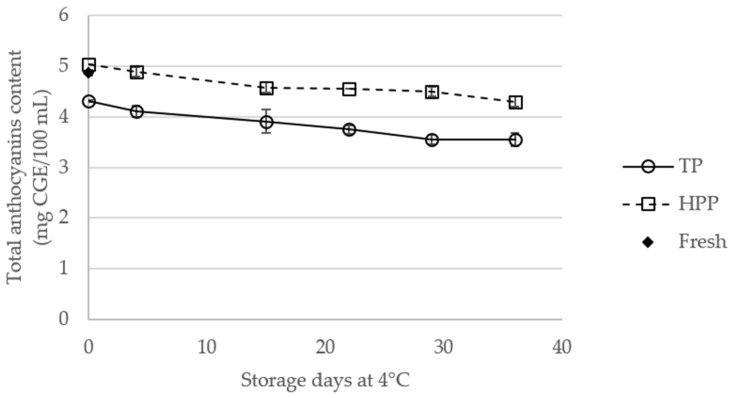
Total anthocyanin content (expressed as cyanidin-3-glucoside equivalents (CGE)) of fresh and thermal pasteurized (TP) and high-pressure processed (HPP) orange juices during storage at 4 °C.

The total anthocyanin contents of fresh and TP and HPP orange juices, during storage, are presented in Figure 3 (the statistical data are available in Supplementary Material, Table S3). On the treatment day, TP juice showed the lowest anthocyanin content (4.31 ± 0.02 mg cyanidin-3-glucoside equivalents (CGE)/100 mL), which might be related to the thermal degradation of these components, while HPP juices showed the highest content (5.03 ± 0.00 mg CGE/100 mL), both variations being statistically significant (*p* < 0.05). During storage, a decrease in anthocyanin content is observed in TP and HPP juices, with lower anthocyanin content in TP juices compared to HPP samples on all sampling days (*p* < 0.05). Degradation of anthocyanins in treated orange juice has been considered as a result of indirect oxidation by phenolic quinones generated by polyphenol oxidase [25]. Notwithstanding, the differences between the HPP and fresh orange juice are only significant after 22 days of storage, in opposition with that verified for TP juices, which present significantly lower anthocyanin content than fresh juice during the entire storage period. 

### 2.4. Effect of Storage of TP and HPP Orange Juice on Total Carotenoid Content

**Figure 4:**
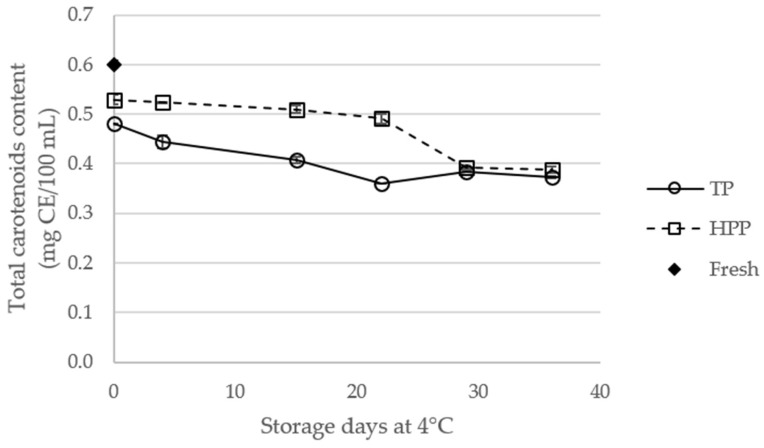
Total carotenoid content (expressed as β-carotene equivalents (CE)) of fresh and thermal pasteurized (TP) and high-pressure processed (HPP) orange juices during storage at 4 °C.

The total carotenoid content of fresh and treated orange juices, during storage, is shown in Figure 4. Carotenoids are one of the most important indicators of orange juice quality, contributing to color, nutritional, and functional properties of the juice [15]. Compared to fresh juice, both TP and HPP treatments significantly decreased (*p* < 0.05) (Table S4) the total carotenoid content by 12% and 20%, respectively. This negative effect of TP and HPP is in agreement with that reported by Vélazquez-Estrada et al. [21] and Bull et al. [26], despite other authors having reported the highest carotenoid content in processed orange juices [18], in particular, in HPP juices [22]. The difference between TP and HPP juices, and fresh juices, were also significant during the storage. In addition, a decrease (*p* < 0.05) of the total carotenoid content of treated juices was also observed during storage. This behavior may be related with the matrix disruption and the polyene chain instability of carotenoids, promoting their isomerization or oxidation [15]. Notwithstanding, HPP juice showed significantly higher carotenoid content than TP juice, during the 36 days of storage (*p* < 0.05). 

### 2.5. Effect of Storage of TP and HPP Orange Juice on Individual Phenolic Compounds

**Figure 5:**
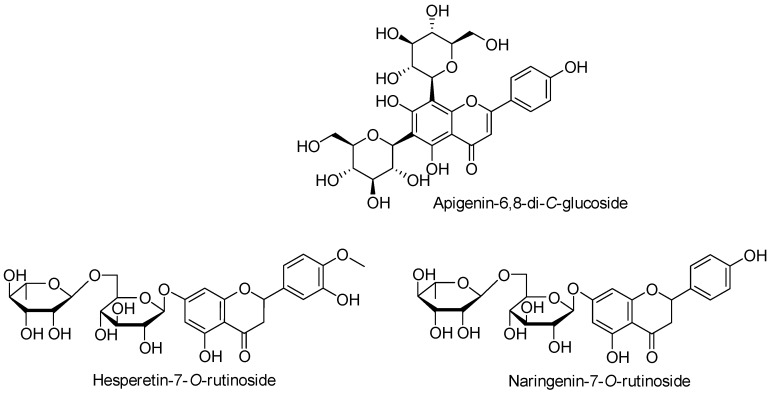
Structures of the major phenolic compounds identified in fresh, thermal pasteurized (TP), and high-pressure processed (HPP) orange juices.

Table 1 summarizes the major phenolic compounds identified in fresh, TP, and HPP orange juices, as well as their retention time, UV absorption maxima, the molecular ion [M − H]^−^, and the corresponding MS^n^ product ions. Compounds were identified by comparing these data with the literature, as indicated in Table 1. Their structures are shown in Figure 5, and the corresponding quantitative data in Table 2.

The phenolic fractions of orange juice samples were shown to be mainly composed of apigenin 6,8-di-*C*-glucoside (vicenin II) (**1**), naringenin 7-*O*-rutinoside (narirutin) (**2**), and hesperetin 7-*O*-rutinoside (hesperidin) (**3**) (Figure 5). As an example, the HPLC-UV chromatogram of HPP orange juice, recorded at 280 nm, is available in Supplementary Material (Figure S1). The high abundance of these components, namely flavonoid derivatives, in orange juices, have already been reported [16,23,30]. 

**Figure 6:**
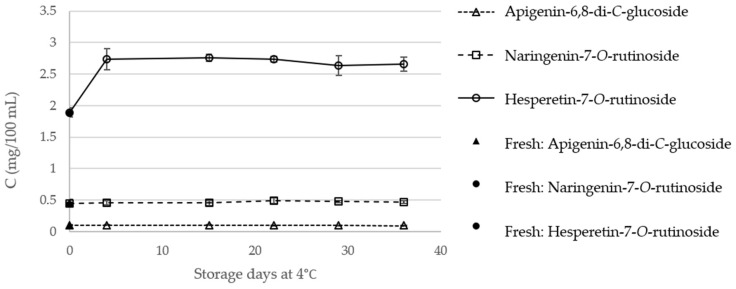
Individual phenolic compounds content (mg/100 mL) of fresh and thermal pasteurized (TP) orange juice during storage at 4 °C.

**Figure 7:**
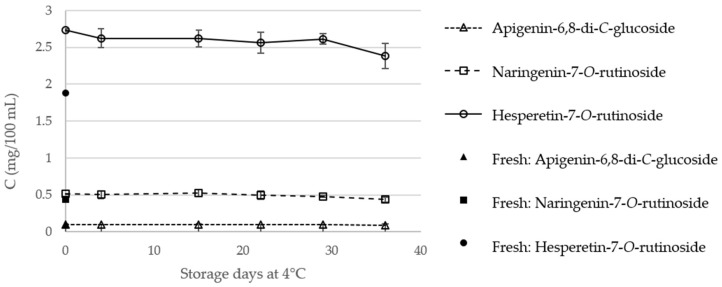
Individual phenolic compounds content (mg/100 mL) of fresh and high-pressure processed (HPP) orange juice during storage at 4 °C.

The content of the major phenolic components, in fresh orange juice and in TP and HPP orange juices, during storage, is presented in Figure 6 and Figure 7, respectively. Regarding fresh, TP, and HPP orange juices, on the day of treatment, the main phenolic components were shown to be hesperetin-7-*O*-rutinoside (**3**), with contents ranging from 1.88 (fresh orange juice) to 2.73 mg/100 mL (HPP orange juices), followed by naringenin-7-*O*-rutinoside (0.44–0.51 mg/100 mL). These contents are considerably lower than those described in the literature for fresh [16] or for processed orange juices [21], which could be related with the orange species or varieties used. Notwithstanding, these differences highlight the need for this comparative study between different treatment methodologies applied on the same raw juice.

The statistical data regarding the individual phenolic compounds content are available in Supplementary Material (Table S5). On the day of treatment, TP did not affect significantly the content of the three analyzed compounds, which is in agreement with that observed for the flavones naringenin and hesperetin, in a study by Sánchez-Moreno et al. [18]. Considering also the day of treatment, HPP was shown to have a positive effect (*p* < 0.05) on the content of hesperetin-7-*O*-rutinoside (**3**), which increased about 46% compared to fresh orange juice, in agreement with the global increments observed above.

The contents of apigenin-6,8-di-*C*-glucoside (**1**) and naringenin-7-*O*-rutinoside (**2**) of TP orange juices was shown to be stable, during the 36 days of storage, in both TP and HPP orange juices. A significant increase in the content of hesperetin-7-*O*-rutinoside (**3**) was also observed for the TP juices at the 4th day of storage, which may be related with the degradation of some components, with the further conversion on hesperetin-7-*O*-rutinoside (**3**). After the 4th day, the content of this flavonoid decreased, although to higher values (2.66 ± 0.11 mg/100 mL) than those observed in the fresh juice (1.88 ± 0.08 mg/100 mL). The storage of HPP orange juices were also shown to decrease the content of hesperetin-7-*O*-rutinoside (**3**). However, at the 36th day, the content of this flavone glycoside (2.38 ± 0.17 mg/100 mL) is still significantly higher than in fresh orange juice (1.88 ± 0.08 mg/100 mL). Therefore, these results reinforce the potential of high pressure as an interesting alternative to preserve, and even enhance, the nutritional properties of orange juice. 

### 2.6. Effect of Storage of TP and HPP Orange Juice on Antioxidant Activity

**Figure 8:**
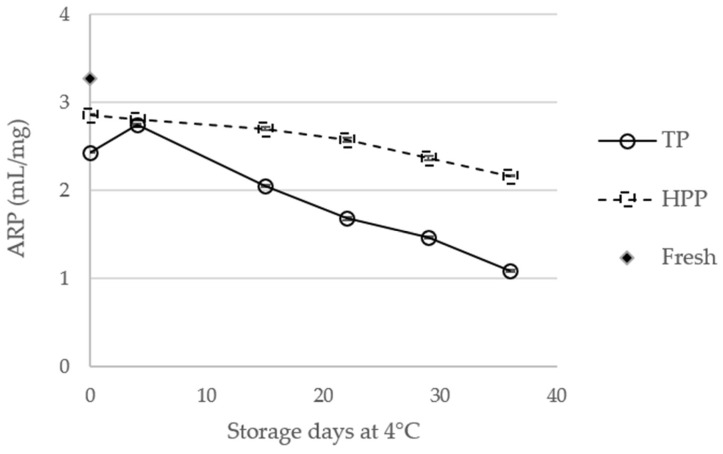
Antioxidant activity, expressed as antiradicalar power (ARP, mL/mg), of fresh orange juice and thermal pasteurized (TP) and high-pressure processed (HPP) orange juices during storage.

The antioxidant activity, expressed as antiradicalar power (ARP), was determined by the 2,2-diphenyl-1-picril-hidrazil (DPPH) assay for fresh orange juice and for TP and HPP orange juices, at the day of the treatment and during storage, as shown in Figure 8. 

Compared to unprocessed orange juice, both TP and HPP were shown to decrease, significantly (*p* < 0.05), the ARP (26% and 13%, respectively). The statistical data are available in Supplementary Material (Table S6). These are interesting results, particularly concerning HPP juices, for which only the carotenoid content was observed to decrease. This allows us to conclude that carotenoids may have an important contribution to the antioxidant activity of the analyzed juices, in opposition to that verified for blood orange juices, in which antioxidant activity was shown to be strictly related with ascorbic acid [31]. 

Other authors have also reported that carotenoids have a negligible contribution for the antioxidant activity of citrus juices [24]. Interestingly, a close correlation was found between total carotenoid content and ARP, particularly for HPP juices (*R*^2^ = 0.9214) The differences between fresh and treated orange juice became higher during storage, particularly for TP juices, which present, at the 36th day of storage, ARP values about 67% lower than fresh juice (compared to the reduction of about 37% for HPP samples). These results show the advantages of HPP compared to TP. In fact, in addition to the higher antioxidant activity observed for HPP juices, compared to TP ones at the day of the treatments, HPP juices also showed lower variations (yet significant) in ARP during storage, the ARP at the 36th day being about 24% lower than in the day of the treatment, while, for TP juices, the difference is about 66%. 

The negative effect of storage of orange juices on their antioxidant activities has been already described [19], although other studies have reported a quasi-stable [23] or even higher [19] antioxidant activity of orange juices during storage. This stability was attributed, by Arena et al. [31], to the ascorbic acid content, which also remained constant during 60 days of storage. However, other authors have ascribed the constant or higher antioxidant activity of foods and beverages during storage, with the formation of Maillard’s reaction products [32]. Notwithstanding, if other components, such as phenolic compounds or carotenoids, have a high contribution to the antioxidant activity, their degradation during storage may also explain the decrease of antioxidant activity, as was observed in this study.

### 2.7. Degradation Kinetics of Stored TP and HPP Orange Juices

The degradation kinetics of ARP, TPC, total flavonoid content, total anthocyanin content, and total carotenoid content of TP and HPP orange juices, was analyzed for zero-, first-, and second-orders, and the best-fitting reaction order coefficients are shown in Table 2. To our knowledge, this is the first study reporting the comparison of kinetic analysis of HPP and TP orange juices during storage. This study is quite important to compare the shelf-life of TP and HPP juices and, therefore, to maximize their nutritional and sensory quality. Most of the responses analyzed followed zero-order kinetics, showing a linear relation with time. However, flavonoid degradation in HPP juices, and anthocyanin degradation in both TP and HPP juices, followed second-order kinetics. Other studies have revealed different degradation kinetics for orange juice components. Agcam et al. [23] reported a second-order kinetic for total phenolics of both pulsed electric field processed or pasteurized orange juices, while Kirca and Cemeroglu [33] showed a first-order kinetic for degradation of anthocyanins of unprocessed blood orange juice and concentrated juice. In this study, it was also demonstrated the high effect of the juice Brix on the degradation kinetic parameters. 

As was described above, the decrease of naringenin-7-*O*-rutinoside, hesperetin-7-*O*-rutinoside and total flavonoids in TP juices, and apigenin-6,8-di-*C*-glucoside in HPP juices, was not observed, therefore, their degradation kinetic parameters were not determined. The kinetic parameter *k* (reaction rate constant) was calculated for the other responses and is presented in Table 2. Total phenolics and total anthocyanins showed *k*-values of 2.3- and 1.6-fold higher, respectively, in TP orange juices. The deterioration of antioxidant activity (ARP) followed different kinetic orders, namely, a zeroth- and first-order in HPP and TP juices, respectively. Notwithstanding, a higher *k*-value was observed for TP juices (about 1.3-fold higher) than in HPP juice.

The half-life time (t_1/2_) of phenolics, flavonoids, and anthocyanins in HPP orange juices were considerably higher than in TP juices, being 65 days for carotenoids and 223 days for anthocyanins. A high half-life time was observed for apigenin-6,8-di-*C*-glucoside in TP juices, however, was not relevant considering the similarity of its initial content in both processed juices, together with its non-degradation in HPP juices. Concerning the antioxidant activity, HPP presented a half-life time 2.7-fold higher than TP juices, increasing it from 29 to 78 days. This highlights the advantages of HPP in the preservation of nutritional and health benefits of orange juice.

## 3. Materials and Methods

### 3.1. Chemicals

Gallic acid, sodium carbonate, ellagic acid, rutin, cyanidin, naringin, potassium acetate, quercetin dihydrate, and 2,2-diphenyl-1-picrylhydrazyl (DPPH) were purchased from Sigma-Aldrich (St. Louis, MO, USA). Folin–Ciocalteu’s reagent was purchased from Merck (Kenilworth, NJ, USA). HCl and aluminum chloride were supplied from Riedel-de Haen (Mexico City, MEX, USA) and M&B (Guildford, UK), respectively. Solvents were purchased from Acros Organics (Geel, Belgium) and Sigma-Aldrich, and were of HPLC grade or analytical grade.

### 3.2. Orange Juice

Orange juice was provided by a local producer of fruit juices (Frubaça—Cooperativa de Hortofruticultores, CRL., Alcobaça, Portugal). Oranges were squeezed on an industrial juice wringer, and then carried through refrigerated tubes to a tank, where they were kept at 4 °C. Orange juice was collected from the same orange juice lot and taken to the laboratory in refrigerated conditions.

### 3.3. TP and HPP Treatment and Storage

On the same day as the orange juice was collected, the treatments were applied. High-pressure processing (HPP) treatment was conducted using industrial equipment (QFP 100L-600, Avure, OH, USA), with a pressure vessel capacity of 100 L, surrounded by a horizontal frame and connected to the water cooling system, with a maximum operating pressure of 600 MPa. After bottling 250 mL, the orange juice was treated at 550 MPa for 70 s at 18 °C. 

For thermal processing (TP), the polyamide/polyethylene (PA/PE-90, Albipack-Packaging Solutions, Águeda, Portugal) plastic bags, with 2 cm of width, containing 5 mL of orange juice, were placed into the water bath at 70 °C. After the internal temperature of sample reached 70 °C (about 70 s after introducing the bags into the water bath), the samples were held at that temperature for 30 s, and then quickly cooled on ice.

Samples of both treatments were stored at 4 °C and were taken for analyses on the following sampling days: 0 (immediately after both treatments), 4, 15, 22, 29, and 36 days. At day 0 of storage, untreated orange juice was also collected for analysis. At each day of analysis, three different samples per treatment were evaluated. 

### 3.4. Determination of TPC

Total phenolic content was determined spectrophotometrically using Folin–Ciocalteu’s assay [34], using gallic acid as standard, and following a modified method described previously [35]. Briefly, 1.5 mL of gallic acid aqueous solutions or diluted orange juice were added to 2.25 mL of distilled water, and 1.5 mL Folin–Ciocalteu’s reagent, previously diluted with water (1:10, *v*/*v*). The mixture was shaken and allowed to stand for 5 min in the dark. Then, 1.5 mL of 6% (*w/v*) sodium carbonate solution was added to the mixture, and the reaction was kept in the dark for 30 min. After incubation, the absorbance was measured at 725 nm, versus the blank, in a 6405 UV/Vis spectrophotometer (Jenway, Staffordshire, UK). A blank was prepared, replacing gallic acid or orange juice by distilled water. Total phenolic content of orange juice was expressed as milligrams of gallic acid equivalents (GAE) per 100 milliliters of juice.

### 3.5. Determination of Total Flavonoid Content

Total flavonoid content was determined using the modified method described previously [36]. Rutin was used as standard. An aliquot (0.2 mL) of the standard solutions (10–100 μg/mL diluted in ethanol), or diluted orange juice samples, were added to 0.6 mL of ethanol, 0.04 mL of 10% aluminum chloride solution, 0.04 mL of 1M potassium acetate solution, and 1.12 mL of distilled water. After incubation at room temperature for 30 min, the absorbance of the reaction mixture was measured at 415 nm with a 6405 UV/Vis spectrophotometer (Jenway, Staffordshire, UK). The amounts of 10% aluminum chloride solution, and the sample, were replaced by the same amount of distilled water in the blank. 

### 3.6. Determination of Total Anthocyanin Content

Total anthocyanin content was determined as described by Rapisarda et al. [37], with some modifications. One milliliter of orange juice was diluted to 10 mL of a 10.15:39.85 (*v*/*v*) mixture of 95% ethanol and 37% HCl. The absorbance was measured at 535 nm in a 6405 UV/Vis spectrophotometer (Jenway, Staffordshire, UK). Total anthocyanin concentration, in mg equivalents of cyanidin-3-glucoside (cy-3-glu) per mL, was calculated according to the Beer–Lambert law, using the molar absorptivity coefficient of 60.45 mL/(mg∙cm).

### 3.7. Determination of Total Carotenoid Content

The total carotenoid concentration was determined following the adapted methods described by George et al. [38] and Rajchl et al. [39]. The orange juice was mixed with acetone (1:8, *v*/*v*) in a SpeedVac tubes, previously wrapped in aluminum paper. Then, samples were left at 4 °C for 30 min, with stirring every 10 min. After incubation, the tubes were placed into the SpeedVac (Univapo Rotational Vacuum Concentrators 100H, Montreal Biotech Inc., Montreal, QC, Canada) for 30–40 min, until complete evaporation of acetone. Petroleum ether (3.50 mL) was added to each SpeedVac tube, following by stirring and incubation for 1 h at 4 °C. Then, 1 mL of distilled water was added to mixture, and centrifugated at 2500 rpm for 5 min in a Compact Tabletop Centrifuge 2010 (Kubota Corporation, Laboratory Centrifuge Japan, Bunkyo-ku, Tokyo, Japan). The absorbance of the supernatant was measured in a 6405 UV/Vis spectrophotometer (Jenway, Staffordshire, UK) at 450 nm, against a blank composed by petroleum ether. Total carotenoid concentration in β-carotene equivalents (CE) was made using the molar absorptivity coefficient of 259.2 mL/(mg∙cm).

### 3.8. Determination of Individual Compound Contents by HPLC-UV-MS^n^

#### 3.8.1. High-Performance Liquid Chromatography (HPLC) Procedure

The major phenolic compounds present in orange juice were identified and quantified by high-performance liquid chromatography (HPLC-UV-MS), as described by Santos et al. [40]. The HPLC system consisted of a variable loop Accela autosampler (200 vial capacity set at 15 °C), an Accela 600 LC pump and an Accela 80 Hz PDA detector (Thermo Fisher Scientific, San Jose, CA, USA). The separation of orange juice compounds was carried out at 25 °C, with a gradient elution program at a flow rate of 0.2 mL/min. The mobile phase consisted of water/acetonitrile (90:10, *v*/*v*) (A) and acetonitrile (B), both with 0.1% of formic acid, applying the following linear gradient: 0–3 min, 0% B; 3–10 min, 0–10% B; 10–30 min, 10–20% B; 30–35 min, 20–25% B; 35–50 min, 25–50% B; 50–55 min, 50–100% B; followed by a ten min re-equilibration time before the next run. Before the injection, orange juice samples were filtered through a 0.2 µm PTFE syringe filter (VWR Internationa l, Radnor, PA, USA). Double online detection was carried out in the diode array detector, at 280 and 365 nm, and UV spectra in a range of 210–600 nm were also recorded. 

#### 3.8.2. ESI-MS^n^ Analysis

HPLC was coupled to an LCQ Fleet ion trap mass spectrometer (ThermoFinnigan, San Jose, CA, USA), equipped with an electrospray ionization source and operating in negative mode. The spray voltage was 5 kV, and the capillary temperature 300 °C. The capillary and tune lens voltage were set at −28 V and −225 V, respectively. Collision-induced dissociation-mass spectrometry (CID-MS^n^) experiments were performed on mass-selected precursor ions in the range of *m*/*z* 100–1000. The isolation width of precursor ions was 1.0 mass units. The scan time was equal to 100 ms, and the collision energy was optimized between 15 and 45 (arbitrary units), using helium as collision gas. The data acquisition was carried out by using Xcalibur^®^ data system (ThermoFinnigan, San Jose, CA, USA). 

#### 3.8.3. Individual Compounds Quantification by HPLC-UV

Calibration curves were obtained by HPLC injection of quercetin dihydrate and naringenin aqueous solutions, with concentration ranges between 5 and 300 µg/mL. The calibration curves and additional relevant data are shown in Supplementary Material (Table S5). Compounds were quantified using the calibration curve of the most similar standard. The concentrations were calculated in triplicate and the mean value and standard deviation computed for each case.

### 3.9. Total Antioxidant Activity by 2,2-Diphenyl-1-Picril-Hidrazil (DPPH) Assay

The antioxidant activity was determined according to the modified method of Kelebek et al. [41] using 2,2-diphenyl-1-picril-hidrazil (DPPH) as a free radical. Briefly, five different aqueous dilution samples of the orange juices (5%, 10%, 15%, 20%, and 30%, *v*/*v*) were firstly prepared. A set of standard DPPH solutions (0–95 µM) in methanol were also prepared. These standard solutions were used to make the calibration curve. An aliquot of 75 µL of diluted orange juice solution was added to 2.93 mL of a 60 µM DPPH solution in methanol, and left to react for 45 min in a dark at room temperature. Finally, the absorbance of each sample was measured at 515 nm using a 6405 UV/Vis spectrophotometer (Jenway, Staffordshire, UK), and using methanol as blank solution. The DPPH concentration in mg/mL (C_DPPH_) in the reaction medium was calculated from the calibration curve with Equation (1). All tests were performed in duplicate.

(1) $$Abs\left(515nm\right)=25.595\times \left(C_{DPPH}\right)-0.0945\;\left(R^{2}=0.9955\right)$$

Effective concentration (EC_50_) was then calculated using the remaining DPPH percentage (% DPPH_remain_), as shown in Equation (2). Antioxidant values were expressed as antiradicalar power (ARP), which is the inverse of EC50 [42] and represents the antioxidant efficiency.

(2) $$\%\;DPPH_{remain}=\frac{CDPPH_{t=0}}{C_{DPPH}}\times 100$$

### 3.10. Statistical Analysis

The effects of treatment method and storage time were tested using SPSS 12.0 for Windows (SPSS Inc., Chicago, IL, USA) in a one-way analysis of variance (ANOVA), followed by a multiple comparisons test (Tuckey’s HSD) to find which samples were significantly different from each other. Differences between treatments were tested at a 0.05 level of significance. All data are expressed as the mean ± standard deviation. All tests were performed in triplicate. 

### 3.11. Kinetic Data Analysis

Kinetic orders, for the degradation of phenolic compounds, flavonoids, anthocyanins, and carotenoids, were determined by first plotting the (parameter), ln(parameter) 1/(parameter) as a function of time (t) for zero-, first-, and second-order kinetics, followed by the selection of the equation fitting with the best linearity (*R*^2^). Half-lives (t_1/2_) were then calculated according to the selected reaction order [43].

## 4. Conclusions

This study aimed to evaluate the effect on bioactive components composition and antioxidant activity, of the storage of TP and HPP orange juices. Comparing the different orange juices at the day of the treatment, no significant differences were observed in the TPC of TP and HPP juices, compared to fresh juices. In comparison with fresh orange juice (untreated), TP was shown to decrease the flavonoid, anthocyanin, and carotenoid contents, while HPP-treated juice showed higher flavonoid content and anthocyanin content, and lower carotenoid content. Three major components were identified in all orange juices, namely, apigenin-6,8-di-*C*-glucoside, naringenin-7-*O*-rutinoside, and hesperetin-7-*O*-rutinoside. A significantly higher content was observed for hesperetin-7-*O*-rutinoside in HPP juice (2.73 mg/100 mL). During storage, a decrease in TPC, and the total anthocyanin and carotenoid contents of both TP and HPP juices, was observed, with HPP juices showing higher anthocyanin and phenolic contents. Storage of treated juices was shown to decrease the content of hesperetin-7-*O*-rutinoside in both TP and HPP juices during storage, however, at the 36th stored day, their contents remained 1.3-fold and 1.4-fold higher in HPP and TP, respectively, than in fresh juice. No significant differences were observed for the other components. Compared to fresh orange juice, both TP and HPP were shown to significantly decrease (26% and 13%, respectively) the antioxidant activity. The differences between fresh and treated orange juice become higher during storage, particularly for TP juices, which present, at the 36th stored day, antioxidant activities about 67% lower than fresh juice. The kinetic analysis of the degradation of TP and HPP orange juices was also studied for the first time. Most of the responses were shown to follow zero-order kinetics. HPP juice showed considerably higher half-life than TP juice for all the studied responses. These clearly demonstrate the advantages of HPP as a promising methodology for orange juice preservation, extending its shelf-life, as well as allowing also for enhancement of its nutritional benefits, increasing the content of some bioactive compounds. 

## Figures and Tables

**Table 1:** Major phenolic compounds found in fresh, TP, and HPP orange juices and the corresponding MS^n^ fragmentation profile.

N.	Rt (min)	λ (nm)	Compound	[M − H]^−^ (*m*/*z*)	MS^n^ Product Ions (*m*/*z*)		Ref.
					MS^2^	MS^3^	
**1**	13.40	235, 271, 333	Apigenin 6,8-di-*C*-glucoside	593	575, 503, 473, 383, 353, 323	[473]: 189, 293	[27,28]
**2**	20.84	234, 283, 329(sh ^i^)	Naringenin 7-*O*-rutinoside	579	271	-	[29]
**3**	23.15	234, 283, 325(sh)	Hesperetin 7-*O*-rutinoside	609	301	283, 256	[29]

^i^ (sh) is used to indicate a shoulder in the UV spectrum.

**Table 2:** Degradation kinetic parameters of TPC, total anthocyanin content, total flavonoid content, total carotenoid content, and ARP for thermal pasteurized (TP) and high-pressure processed (HPP) orange juices stored for 36 days.

Parameter	Condition	Reaction Order	*k*-Value ^i^	*R* ^2^	t_1/2 (days)_
TPC	TP	0	0.656	0.997	70
	HPP	0	0.282	0.879	154
Total flavonoids	TP	-	-	-	-
	HPP	2	0.2 × 10^−3^	0.955	76
Total anthocyanins	TP	2	1.4 × 10^−3^	0.968	168
	HPP	2	0.9 × 10^−3^	0.949	223
Total carotenoids	TP	0	2.9 × 10^−3^	0.795	79
	HPP	0	4.2 × 10^−3^	0.834	65
Apigenin-6,8-di-*C*-glucoside	TP	0	0.1 × 10^−3^	0.657	528
	HPP	-	-	-	-
Naringenin-7-*O*-rutinoside	TP	-	-	-	-
	HPP	0	1.8 × 10^−3^	0.644	148
Hesperetin-7-*O*-rutinoside	TP	-	-	-	-
	HPP	0	6.9 × 10^−3^	0.685	197
ARP	TP	1	2.36 × 10^−2^	0.937	29
	HPP	0	1.85 × 10^−2^	0.945	78

^i^*k*-value expressed as mg∙100 mL^−1^∙day^−1^, for 0th-order kinetics; day^−1^, for 1st-order kinetics; 100 mL∙mg^−1^∙day^−1^, for 2nd-order kinetics.

## Supplementary Materials

The following are available online, Table S1: TPC (mg gallic acid equivalents/100 mL) of fresh, TP, and HPP orange juices and respective statistical data, Table S2: Total flavonoid content (mg rutin equivalents/100 mL) of fresh, TP, and HPP orange juices and respective statistical data, Table S3: Total anthocyanin content (mg cyanidin-3-glucoside equivalents/100 mL) of fresh, TP, and HPP orange juices and respective statistical data, Table S4. Total carotenoid content (mg β-carotene equivalents/100 mL) of fresh, TP, and HPP orange juices and respective statistical data, Table S5. Individual compounds content (mg/L) found in fresh and in thermal pasteurized (TP) and high-pressure processed (HPP) orange juices during storage, Table S6. Antioxidant activity expressed as antiradicalar power (ARP) (mL/mg) of fresh, thermal pasteurized (TP) and high-pressure processed (HPP) orange juices and respective statistical data, Table S7. Calibration data used for the HPLC-UV quantification of major components of fresh, thermal pasteurized (TP) and high-pressure processed (HPP) orange juices, Figure S1. HPLC-UV chromatogram of HPP orange juice at day of the treatment, recorded at 280 nm (1-Apigenin-6,8-di-*C*-glucoside, 2-Naringenin-7-*O*-rutinoside, 3-Hesperetin-7-*O*-rutinoside).
