# Supplementary material for: Long-Term Effect on Bioactive Components and Antioxidant Activity of Thermal and High-Pressure Pasteurization of Orange Juice
Source: Molecules. 2018 Oct 20;23(10):2706. doi: 10.3390/molecules23102706 (PMC6222786; doi:10.3390/molecules23102706)
Supplement: Supplementary file 1 [file molecules-23-02706-s001.pdf]

Supplementary Material

Table S1. TPC (mg gallic acid equivalents/100 mL) of fresh, thermal pasteurized (TP) and high-pressure processed (HPP) orange juices and respective statistical data.

Time of storage (days)	Fresh[a]	TP	HPP
0	89.00±5.40 A	90.40±4.31 eA	84.80±4.70 aA
4	A	89.30±0.81 deA	86.90±5.20 aA
15	A	81.80±4.05 cdA	83.90±1.90 abA
22	B	76.60±2.72 bcA	82.20±2.20 abAB
29	B	72.30±2.74 abA	78.20±1.60 abA
36	C	67.40±1.24 aA	75.70±0.70 bB

Results represent average±standard deviation (n=3). Different non-capital letters in the same treatment during storage indicate significant differences ($p<0.05$); Different capital letters in the same day of storage indicate significant differences between fresh, TP and HPP juices ($p<0.05$); [a] Capital letters in fresh column from 4th to 36th day indicates the statistical result of the comparison between fresh orange juice with TP and HPP juices at each sampling day.

Table S2. Total flavonoids content (mg rutin equivalents/100 mL) of fresh, thermal pasteurized (TP) and high-pressure processed (HPP) orange juices and respective statistical data.

Time of storage (days)	Fresh[a]	TP	HPP
0	57.3±1.0 B	44.8±1.0 cA	70.8±0.3 eC
4	A	60.8±2.0 aB	58.1±0.8 dAB
15	A	57.3±1.8 abA	53.5±0.4 cB
22	A	53.9±6.1 abA	48.7±2.8 bA
29	A	61.2±3.6 aB	45.8±2.1 abB
36	C	49.4±2.8 bcB	43±0.1 aA

Results represent average±standard deviation (n=3). Different non-capital letters in the same treatment during storage indicate significant differences ($p<0.05$); Different capital letters in the same day of storage indicate significant differences between fresh, TP and HPP juices ($p<0.05$); [a] Capital letters in fresh column from 4th to 36th day indicates the statistical result of the comparison between fresh orange juice with TP and HPP juices at each sampling day.

Table S3. Total anthocyanins content (mg cyanidin-3-glucoside equivalents/100 mL) of fresh, thermal pasteurized (TP) and high-pressure processed (HPP) orange juices and respective statistical data.

Time of storage (days)	Fresh[a]	TP	HPP
0	4.87±0.05 B	4.31±0.02 dA	5.03±0.01 cC
4	B	4.11±0.11 cdA	4.89±0.12 cB
15	B	3.91±0.23 bcA	4.58±0.11 aB
22	C	3.75±0.08 abA	4.56±0.04 aB
29	C	3.55±0.09 aA	4.50±0.09 bcB
36	C	3.55±0.13 aA	4.30±0.10 bcB

Results represent average±standard deviation (n=3). Different non-capital letters in the same treatment during storage indicate significant differences ($p<0.05$); Different capital letters in the same day of storage indicate significant differences between fresh, TP and HPP juices ($P<0.05$); [a] Capital letters in fresh column from 4th to 36th day indicates the statistical result of the comparison between fresh orange juice with TP and HPP juices at each sampling day.

Table S4. Total carotenoids content (μg *β*-carotene equivalents/100 mL) of fresh, thermal pasteurized (TP) and high-pressure processed (HPP) orange juices and respective statistical data.

Time of storage (days)	Fresh[a]	TP	HPP
0	601.0±1.4 C	480.0±1.1 eA	529.0±10.0 eB
4	C	444.0±12.6 dA	524.0±1.1 bcB
15	C	408.0±5.9 cA	510.0±8.4 bB
22	C	360.0±0.0 bA	491.0±8.9 dB
29	B	384.0±1.1 abA	392.0±1.9 aB
36	C	373.0±2.2 aA	388.0±5.9 aB

Results represent average±standard deviation (n=3). Different non-capital letters in the same treatment during storage indicate significant differences ($p<0.05$); Different capital letters in the same day of storage indicate significant differences between fresh, TP and HPP juices ($p<0.05$); [a] Capital letters in fresh column from 4th to 36th day indicates the statistical result of the comparison between fresh orange juice with TP and HPP juices at each sampling day.

Table S5. Individual compounds content (mg/L) found in fresh and in thermal pasteurized (TP) and high-pressure processed (HPP) orange juices during storage.

Compound	Storage days	Fresh[a]	TP	HPP
Apigenin-6,8-di-*C*-glucoside[b]	0	1.04±0.03 A	1.05±0.02 aA	0.97±0.05 aA
	4	B	1.05±0.05 aA	1.01±0.01 aA
	15	B	1.05±0.02 aA	1.01±0.01 aA
	22	B	1.03±0.01 aAB	0.99±0.02 aA
	29	B	1.04±0.01 aA	0.98±0.03 aA
	36	B	1.01±0.04 aA	0.92±0.13 aA
Naringenin-7-*O*-rutinoside[c]	0	4.36±1.70 A	4.52±0.60 aA	5.10±0.09 aA
	4	A	4.57±0.35 aA	5.03±0.52 aA
	15	A	4.53±0.39 aA	5.23±0.45 aA
	22	A	4.95±0.44 aA	4.92±0.56 aA
	29	A	4.76±0.19 aA	4.78±0.15 aA
	36	A	4.70±0.24 aA	4.36±0.31 aA
Hesperetin-7-*O*-rutinoside[c]	0	18.76±0.79 A	18.92±0.74 bA	27.34±0.21 bB
	4	B	27.35±1.66 aA	26.24±1.30 abA
	15	B	27.60±0.57 aA	26.20±1.05 abA
	22	B	27.34±0.47 aA	25.60±1.41 abA
	29	B	26.37±1.55 aA	26.13±0.71 abA
	36	B	26.61±1.12 aA	23.80±1.69 aA

Results represent average±standard deviation (n=3). Different non-capital letters in the same treatment during storage indicates significant differences ($p<0.05$). Different capital letters in the same day of storage indicate significant differences between fresh, TP and HPP orange juices ($p<0.05$). [a]Capital letters in fresh column at days 4 to 36 indicate statistical data used to compared TP and HPP orange juices. [b]quantified with quercetin at 365 nm. [c]quantified with naringenin at 280 nm.

Table S6. Antioxidant activity expressed as antiradicalar power (ARP) (mL/mg) of fresh, thermal pasteurized (TP) and high-pressure processed (HPP) orange juices and respective statistical data.

Time of storage (days)	Fresh[a]	TP	HPP
0	3.270±0.007C	2.433±0.007 fA	2.860±0.009 eB
4	B	2.750±0.007 eA	2.800±0.001 fA
15	C	2.048±0.008 dA	2.698±0.020 dB
22	C	1.679±0.020 cA	2.577±0.022 cB
29	B	1.467±0.009 bA	2.368±0.020 bB
36	C	1.088±0.010 aA	2.164±0.002 aB

Results represent average±standard deviation (n=3). Different non-capital letters in the same treatment during storage indicate significant differences ($p<0.05$); Different capital letters in the same day of storage indicate significant differences between fresh, TP and HPP juices ($p<0.05$); [a] Capital letters in fresh column from 4th to 36th day indicates the statistical result of the comparison between fresh orange juice with TP and HPP juices at each sampling day.

Table S7. Calibration data used for the HPLC-UV quantification of major phenolic components of fresh, thermal pasteurized (TP) and high-pressure processed (HPP) orange juices

Compound	*λ* (nm)	Concentration range (μg mL^{-1})	Calibration curve[a]	R^2	LOD[b] (μg mL^{-1})	LOQ[c] (μg mL^{-1})
**Naringenin**	280	5-200	y=623591x+1103161	0.997	38.81	129.36
**Quercetin**	365	10-100	y=665124x+1013845	1.000	1.16	3.88

[a] y=peak area, x=concentration in μg mL^{-1}; [b]LOD-limit of detection; [c]LOQ-limit of quantification

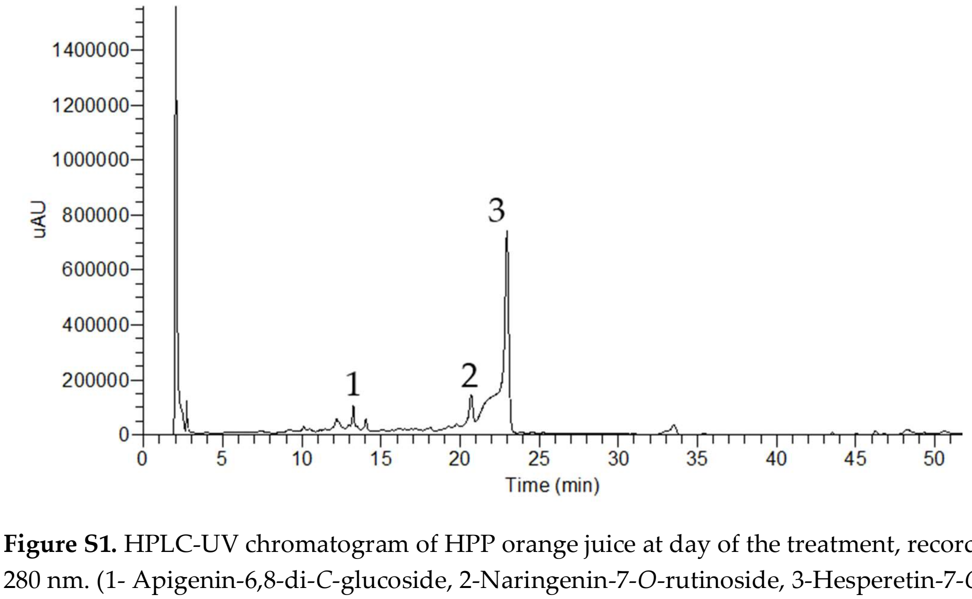

Figure S1. HPLC-UV chromatogram of HPP orange juice at day of the treatment, recorded at 280 nm. (1- Apigenin-6,8-di-*C*-glucoside, 2-Naringenin-7-*O*-rutinoside, 3-Hesperetin-7-*O*-rutinoside)
